# Rack1 Controls Parallel Fiber–Purkinje Cell Synaptogenesis and Synaptic Transmission

**DOI:** 10.3389/fncel.2019.00539

**Published:** 2019-12-17

**Authors:** Haihong Yang, Chaojuan Yang, Qian Zhu, Mengping Wei, Ying Li, Juanxian Cheng, Fengjiao Liu, Yan Wu, Jiyan Zhang, Chen Zhang, Haitao Wu

**Affiliations:** ^1^Department of Neurobiology, Beijing Institute of Basic Medical Sciences, Beijing, China; ^2^Department of Anesthesiology, The General Hospital of Western Theater Command, Chengdu, China; ^3^Department of Neurobiology, School of Basic Medical Sciences, Capital Medical University, Beijing, China; ^4^Department of Neuroimmunology and Antibody Engineering, Beijing Institute of Basic Medical Sciences, Beijing, China; ^5^Chinese Institute for Brain Research, Beijing, China; ^6^Key Laboratory of Neuroregeneration, Co-innovation Center of Neuroregeneration, Nantong University, Nantong, China

**Keywords:** parallel fiber, Purkinje cell, Rack1, LTD, synaptic plasticity

## Abstract

Purkinje cells (PCs) in the cerebellum receive two excitatory afferents including granule cells-derived parallel fiber (PF) and the climbing fiber. Scaffolding protein Rack1 is highly expressed in the cerebellar PCs. Here, we found delayed formation of specific cerebellar vermis lobule and impaired motor coordination in PC-specific Rack1 conditional knockout mice. Our studies further revealed that Rack1 is essential for PF–PC synapse formation. In addition, Rack1 plays a critical role in regulating synaptic plasticity and long-term depression (LTD) induction of PF–PC synapses without changing the expression of postsynaptic proteins. Together, we have discovered Rack1 as the crucial molecule that controls PF–PC synaptogenesis and synaptic plasticity. Our studies provide a novel molecular insight into the mechanisms underlying the neural development and neuroplasticity in the cerebellum.

## Introduction

The multiple functions of the brain depend on the precise communication between distinct types of neurons. Communication between neurons is achieved at synapses by the process of synaptic transmission within neuronal networks (Jones, 2005; Cohen and Greenberg, 2008). Therefore, deciphering the molecular mechanisms underlying the development and function of synapses is the key aspect of cellular and molecular neuroscience. Owing to the unique patterned foliation, typical “three-layer” cortex, and relatively simple cell types, the cerebellum serves as an ideal model for studying the development and function of synapses and brain circuits (Middleton and Strick, 1998).

As the only efferent neurons in the cerebellar cortex, Purkinje cells (PCs) receive two types of excitatory synaptic inputs: climbing fibers and parallel fibers (PFs), and integrate cortical information for the deep cerebellar nuclei (Brown et al., 2012; Duguid et al., 2015; Nietz et al., 2017). The interaction between climbing fiber and PF inputs into PCs is critical for motor learning (Ito, 2002, 2006). Particularly, PF–PC synapses are the fundamental connections in the cerebellar cortex, which play an essential role in cerebellar synaptic plasticity and motor coordination (Guan et al., 2014). PF–PC synapses are generally viewed as a uniform population with homogeneous postsynaptic properties. PCs express several types of ionotropic and metabotropic glutamate receptors (mGluRs) including α-amino-3-hydroxy-5-methyl-4-isoxazole propionic acid receptor (AMPA), *N*-methyl-D-aspartate receptor (NMDAR), mGluR, etc., which comprise different combinations of receptor subunits (Okubo et al., 2004; Jin et al., 2007). Several works have demonstrated the formation and maintenance of PF–PC synapses depending on distinct sets of molecules and synaptic organizers. Till now, the identification of novel molecules that modulate PF–PC synapse formation and synaptic transmission remains a critical open question.

The receptor for activated C kinase 1 (Rack1) is a multifaceted scaffolding protein with seven conserved WD40-repeat domains, which was originally identified as an anchoring protein for the conventional protein kinase C (PKC) (Adams et al., 2011; Li and Xie, 2015). Increasing evidence suggests that Rack1 was involved in the regulation of neural development and brain functions (Wang and Friedman, 2001; McGough et al., 2004; Sklan et al., 2006; Wehner et al., 2011; Kershner and Welshhans, 2017a, b). Our previous work has demonstrated that Rack1 controlled the mammalian cerebellar development by opposite regulation of Wnt/β-catenin and Sonic hedgehog (Shh) signaling pathways in neural stem cells and granule cell progenitors (G), respectively (Yang et al., 2019). Previous studies and our latest work both indicate the enriched expression of Rack1 in the cerebellum, especially in PCs (Ashique et al., 2006; Yang et al., 2019). Interestingly, Rack1 binds and negatively regulates NMDAR subunit NMDAR subtype 2B by inhibition of non-receptor protein tyrosine kinase Fyn phosphorylation in the hippocampus (Yaka et al., 2002; Thornton et al., 2004). However, whether Rack1 in PCs participates in PF–PC synapse formation and function is still elusive.

To understand the role of Rack1 in the regulation of cerebellar synaptogenesis and long-term depression (LTD) at PF–PC synapses, we first generated PC-specific Rack1 knockout mice. Morphological and ultrastructural studies demonstrate that Rack1 mutant mice exhibit delayed formation of cerebellar vermis specifically to lobule VII as well as significantly decreased number of PF–PC synapses. Induction of PF–PC LTD was also severely impaired in Rack1 mutant mice. Consistently, Rack1 mutant mice also showed significant motor coordination defects. Together, our studies demonstrated that Rack1 in PCs is responsible for PF–PC synaptogenesis and synaptic transmission.

## Materials and Methods

### Animals

The *Rack1*^*F/F*^ lines were generated as previously described, in which exon 2 of *Rack1* gene was flanked by loxP sites (Zhao et al., 2015). Homozygous *Rack1*^*F/F*^ mice were crossed with mice expressing a transgene encoding Cre recombinase driven by *Pcp2* promoter (Barski et al., 2000). Conditional knockout mice were generated by the second generation, and *Rack1*^*F/F*^ littermates served as wild-type controls. All experiments with animals were performed in accordance with protocols approved by the Institutional Animal Care and Use Committee of Beijing Institute of Basic Medical Sciences. Mice were housed in specific pathogen-free conditions with 12/12-h light/dark cycles at Beijing Institute of Basic Medical Sciences.

### Immunofluorescent Staining

It was performed as previously described (Wu et al., 2015; Yang et al., 2019). Briefly, frozen sections were washed 10 min with 0.5% phosphate-buffered saline with Tween 20 (PBS-T) for three times and then blocked with 3% bovine serum albumin for 1 hr. After that, sections were incubated overnight at 4°C with the primary antibodies as follows: Calbindin (C9848, Sigma, 1:400), NeuN (MAB377, Millipore, 1:400), brain lipid binding protein (BLBP) (ab32423, Abcam, 1:500), Rack1 (R1905, Sigma, 1:400). The sections were washed 10 min with 0.5% PBS-T for three times again and subsequently subjected to Alexa Fluor-conjugated secondary antibodies (Biotium, 1:500). Nuclear staining was visualized with a mounting medium with 4′,6-diamidino-2-phenylindole (ZSGB-BIO). All images were taken from a laser scanning confocal microscope (Olympus FV1200) and then were processed and analyzed by FV10-ASW or Image Pro Plus 6.0 software.

### Nissl Staining

The sections (12 μm) of cerebellum mounted on gelatin-coated slides were washed 10 min with 0.5% PBS-T for three times and then immersed into 0.5% tar-violet solution for 20 min. The slices were then quickly rinsed in distilled water and differentiated in 95% ethanol for 2 min. Then, they were dehydrated in 75% ethanol twice, 3 min each. Finally, the slices were sealed with neutral resin.

### Transmission Electron Microscopy

The cerebellum were taken from mice at postnatal day 21 (P21) and then fixed in 2% formaldehyde and 2.5% glutaraldehyde in 0.1 M sodium cacodylate buffer (pH 7.4). After 12 h, the cerebellum were washed thoroughly and soaked in 0.1 M sodium dimethylarsenate buffer. The cerebellum was embedded in 4% agar and trimmed with a conventional microtome. After that, sections were fixed in 1% osmium tetroxide/1.5% potassium ferrocyanide solution for 1 h, washed three times in distilled water, incubated in 1% uranium peroxide acetate for 1 hr, washed twice in distilled water, and then dehydrated with gradient alcohol (50, 70, and 90%, 10 min each time; 100%, 10 min twice). Finally, the samples were incubated with propylene oxide for 1 h and then percolated overnight in a 1:1 mixture of propylene oxide and Epon (TAAB, United Kingdom). Next day, the samples were embedded in Epon and polymerized for 48 h at 60°C. Ultrathin sections (about 60–80 nm) were cut on Reichert Ultracut-S microtome sagittally and picked up on to a copper mesh stained with lead citrate. The formation of PF–PC synapses was observed by a transmission electron microscopy (Hitachi, H-7650) with an AMT 2k CCD camera.

### Golgi Staining

Golgi staining was administrated with FD Rapid GolgiStain^TM^ Kit (PK401). Briefly, mice were deeply anesthetized before killing, and cerebellum was removed from the skull as quickly as possible, but handled carefully to avoid damaging or pressing of the tissue. Tissue was immersed in the impregnation solution made by mixing equal volumes of solutions A and B and was put aside at room temperature for at least 2 weeks in the dark, and then, tissue was transferred into solution C followed by storage at room temperature in the dark for 72 h. The 100-μm sections were cut on a vibrating slicer (Leica, VT1200 S). Each section was mounted on gelatin-coated microscope slides with solution C and dried naturally at room temperature. Sections were rinsed in double-distilled water twice, 4 min each, and then placed in a mixture consisting of one part solution D, one part of solution E, and two parts of double-distilled water for 10 min. Sections were dehydrated in 50, 75, 95, and 100% ethanol successively, 4 min each. Lastly, sections were cleared in xylene for three times, 4 min each, and finally sealed with neutral resin.

### Electrophysiology

#### Brain Slice Preparation

At 21 days, mice were decapitated, and the brain was removed to an ice-cold solution containing 213 mM sucrose, 26 mM NaHCO_3_, 10 mM glucose, 5 mM MgCl_2_, 3 mM KCl, 1 mM NaH_2_PO_4_, and 0.5 mM CaCl_2_. Sagittal slices of cerebellar vermis (250 μm) were prepared using a vibrating blade microtome (VT-1200s, Leica) and were incubated in artificial cerebrospinal fluid containing 125 mM NaCl, 26 mM NaHCO_3_, 10 mM glucose, 5 mM KCl, 2.6 mM CaCl_2_, 2 mM NaH_2_PO_4_, and 1.3 mM MgCl_2_, at a pH of 7.3–7.4, bubbled with 95% O_2_ and 5% CO_2_, for 1 h at room temperature.

#### Whole-Cell Recordings

Whole-cell recordings were obtained with an EPC10 Patch Clamp Amplifier (HEKA, Lambrecht, Germany). Microelectrodes filled with internal solution (3–4 MΩ) were used. The internal solution contained 135 Cs-methanesulfonate, 10 CsCl, 10 4-(2-hydroxyethyl)-1-piperazineethanesulfonic acid, 0.2 ethylene glycol tetraacetic acid, 4 adenosine 5’-triphosphate disodium salt trihydrate, and 0.4 guanosine 5’-triphosphate sodium salt hydrate, pH 7.3, osmolality of 290. For miniature excitatory postsynaptic current (mEPSC) recordings, the slice was then transferred to a chamber perfused with artificial cerebrospinal fluid containing 50 μM picrotoxin (PTX) and 1 μM tetrodotoxin. Evoked EPSCs were pharmacologically isolated by adding 50 μM PTX to the bath solution. The stimulus was delivered to PFs through a concentric bipolar electrode (CBBEB75, FHC, Bowdoin, ME, United States). For LTD of PF-EPSCs recordings, we recorded the baseline for 10 min and then applied five pulse at 100-Hz stimulus and depolarizing the neurons to 0 mV for 100 ms while clamping the cell (30 pulse, 0.5 Hz), followed by 35 min of recording (Zhou et al., 2017). Synaptic responses were collected every 15 s. Somatic whole-cell current-clamp recordings were obtained from PCs in lobule VI or VII of the cerebellar vermis, and series resistances of >20 MΩ were rejected. The electrophysiological data were analyzed using Igor 4.0 (WaveMetrics), and Prism 5 (GraphPad Software).

### Isolation of the Postsynaptic Density Fraction

Cerebellum was removed on ice and placed in a homogenate tube. Homogenate buffer (0.5 g tissue/5 ml homogenate, 0.32 M sucrose, 10 mM 4-(2-hydroxyethyl)-1-piperazineethanesulfonic acid–NaOH, pH 7.4) was added and placed under an electric homogenizer for 20 times. The whole protein fraction was centrifuged at 1,000 × *g*/4°C for 10 min. Then, 4 ml of 1.2 M sucrose solution was added into the ultracentrifuge tube in advance, and the previously obtained supernatant was poured in the top. The ultracentrifuge tube was centrifuged at 160,000 × *g*/4°C for 15 min. The synaptic layer (between 1.2 M sucrose solution and homogenate buffer) was carefully aspirated, and 4 ml of homogenate buffer was added to mix it well. Four milliliters of 0.8 M sucrose solution was added into the ultracentrifuge tube, and the solution obtained in the previous step was slowly added above the 0.8 M sucrose solution. Similarly, the ultracentrifuge tube was centrifuged at 160,000 × *g*/4°C for 15 min. The supernatant was discard, and the pellet was resuspend with 1.6 ml of the resuspension buffer (0.5% Triton X-100, 0.16 M sucrose, 6 mM Tris–HCl, pH 8.1). The synaptic component was centrifuged at 32,800 × *g* for 20 min. The supernatant was thrown away, and 0.4 ml resuspension buffer was added to the pellet to centrifuge for 1 h at 200,000 × *g*. The precipitate obtained after centrifugation was the postsynaptic density (PSD) component.

### Western Blot

The experiments were performed as previously described (Wu et al., 2012). Briefly, PSD fraction isolated from cerebellum tissues was supplemented with 1 × protease and phosphatase inhibitor mixture. Protein concentration was measured using the BCA Protein Assay Kit. Samples (20–50 μg, including 5 μl of prestained protein standards) were loaded into the sodium dodecyl sulfate–polyacrylamide gel electrophoresis gel, and electrophoresis was conducted at constant voltage (120 V) at 4°C and then transferred to polyvinylidene difluoride membranes. The membranes were then blocked with 5% skim milk in 0.1% Tris–buffered saline/Tween-20 for 1 h and incubated overnight at 4°C with indicated primary antibodies. In each experiment, horseradish peroxidase and enhanced chemiluminescence were used to image protein bands on film. The film signal was electronically scanned and statistically analyzed by Image Pro Plus software.

### Behavioral Tests

#### Balance Beam

The device consists of a strong light on the starting side and a safety platform (RWD, R-LBB) on the dark side. Each mouse was acclimated three times with an interval of 10 min before the formal test. The time of mice passing through the 50-cm long balance beam was recorded.

#### Accelerating Rotarod

Following adaptation to the stick (Ugo Basile, 47650), mice were measured every 8 h for eight consecutive times. In each test, the speed was accelerated from 4 to 60 rpm over a 5-min period, and the deadline was 300 s.

#### Open Field

To analyze general locomotion and exploratory behavior in a novel environment, the open field test was performed. Open field apparatus comprised of a transparent plexiglas (40 cm × 40 cm × 40 cm) arena with a white floor virtually (SLY-ETS) divided into two zones: periphery and center. Every mouse was able to explore the novel environment for 5 min. Total distance and center distance traveled by the animal were calculated and analyzed, respectively. Room illumination was kept at 60 lx. Mice position were determined by automatic video tracking (ANY-maze technology).

### Morphometric Analysis of Cerebellar Lobules and PCs

Serial cerebellar coronal cryostat sections were stained with cresyl violet. Golgi-stained brain slices of the whole PCs were taken with an Olympus microscope. One-micrometer-spaced Z-stack brightfield images for dendritic spines were taken with an Olympus BX60 microscope with Axiocam MRc Zeiss camera and Axiovision 4.8 Software (Zeiss, Germany). All images are processed and quantified using Adobe Photoshop CS6 version and ImageJ Software. Spine morphology was determinated based on previous studies (Lee et al., 2004). Spine density was evaluated as the relative spine number over 10-μm dendritic fragments with NeuronStudio software.

### Statistical Analysis

The data between the two independent groups were shown as mean ± SEM of at least three independent experiments. *p*-values were determined by Student’s *t*-test or non-parametric test, and *p* < 0.05 was considered statistically significant.

## Results

### Ablation of *Rack1* in PCs Delays Cerebellar Lobule VII Formation

**FIGURE 1:**
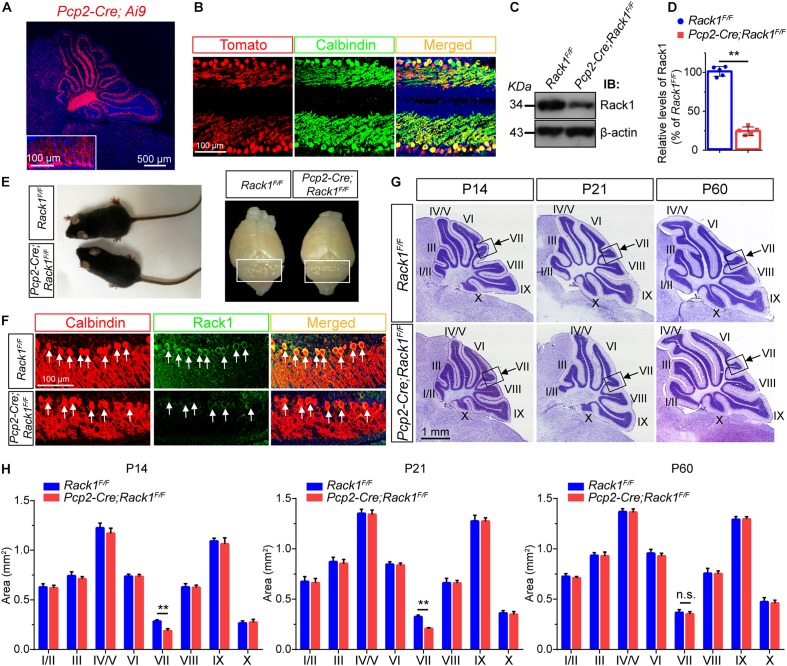
Ablation of *Rack1* in Purkinje cells (PCs) delays cerebellar lobule VII formation. **(A)** Ai9 reporter mice showed that *Pcp2*-Cre is specifically expressed in the PC layer of cerebellar cortex. Scale bar = 500 μm. **(B)** The expression of *Pcp2*-Cre was determined in the offspring of *Pcp2*-Cre;Ai9 mice. Cerebellar sections were counterstained with Calbindin antibody. Scale bar = 100 μm. **(C)** Representative Western blot shows the expression of Rack1 in the postsynaptic density (PSD) fraction of cerebellar lysates from P7 wild-type and *Pcp2-Cre*;*Rack1*^*F/F*^ mutant mice. **(D)** Quantitative analysis of Western blot displays significant decreased expression of Rack1 in *Pcp2-Cre*;*Rack1*^*F/F*^ mutants compared to control littermates. Mean ± SEM, ^∗∗^*p* = 0.0009, *n* = 5. **(E)** The body size of *Pcp2-Cre*;*Rack1*^*F/F*^ mutant mice was indistinguishable compared to control littermates at P30. **(F)** Coimmunofluorescent staining with Rack1 and Calbindin antibodies show the significantly decreased expression of Rack1 in Purkinje cells in *Pcp2-Cre*;*Rack1*^*F/F*^ mutants compared to control littermates. Scale bar = 100 μm. **(G)** Nissl staining of sagittal sections of the cerebellar vermis shows specific deficiency in cerebellar lobule VII foliation in *Pcp2-Cre*;*Rack1*^*F/F*^ mutants compared to control littermates at P14, P21 but not at P60. Scale bar = 1 mm. **(H)** Quantitative analysis of the area of each individual lobule of the vermis in *Pcp2-Cre*;*Rack1*^*F/F*^ mutant mice and control littermates at indicated developmental stages. ^∗∗^*p* = 0.007 and 0.008, at P14 and P21, respectively; *p* = 0.55 at P60, *n* = 5, n.s. = not significant.

Expression of Rack1 protein was fairly rich in mice at birth but decreased gradually at approximately postnatal day P14 and remained constant thereafter (Yang et al., 2019). Immunofluorescent colocalization revealed that Rack1 protein was mainly expressed in PCs at P21 cerebellum. To investigate the potential function of Rack1 in PCs *in vivo*, the conditional knockout mice were generated with hybridization between *Pcp2-Cre* transgenic lines and *Rack1* loxP mice. At first, the *Pcp2-Cre* recombinase was visualized by Ai9 reporter mice. It exhibited that abundant *Pcp2-Cre* recombinase was specifically expressed in PCs (Figures 1A,B). Selective deletion of *Rack1* in PCs were confirmed by both Western blot from isolated synaptic fraction (Figure 1C) and immunofluorescence staining (Figure 1F). It showed robust Rack1 protein decline in Rack1 mutants (Figure 1D, 32.8 ± 1.96 in mutant vs. 100.0 ± 2.78 in control, *p* = 0.0009, *n* = 5). In general, *Rack1* mutant mice appeared normal at P21, as shown by similar body weight and cerebellar surface fissure compared to wild-type littermates (Figure 1E).

Next, we further precisely examined the cerebellar size by quantitative histological analysis. Nissl staining of cerebellar sections indicated that the foliation of *Pcp2-Cre;Rack1^*F/F*^* mutant mice was similar to that of the control littermates postnatally except for lobule VII until P60 (Figure 1G). Sagittal sections of cerebellar vermis indicates that the area of lobule VII but not other lobules was significantly smaller in *Pcp2-Cre;Rack1^*F/F*^* mutant mice compared to control littermates at P14 (0.18 ± 0.02 mm^2^ in mutants vs. 0.29 ± 0.02 mm^2^ in wild-type controls, *p* = 0.007, *n* = 5) and P21 (0.21 ± 0.05 mm^2^ in mutants vs. 0.31 ± 0.02 mm^2^ in wild-type controls, *p* = 0.008, *n* = 5), but not P60 (0.33 ± 0.02 mm^2^ in mutants vs. 0.35 ± 0.02 mm^2^ in wild-type controls, *p* = 0.55, *n* = 5), suggesting that the ablation of *Rack1* in PCs causes the delayed foliation and morphogenesis most specifically restricted to lobule VII, but not other lobules in vermis (Figure 1H). Interestingly, it should be noted that the alterations are only restricted to the vermis subregion but not other cerebellar hemispheres for some unknown reason. Since, the expansion of GNPs is crucial for cerebellar foliation formation, this phenotype is probably due to defects in delayed GNPs proliferation and migration at specific subregion in mutant mice.

### Impaired Motor Coordination and Hyperactivity in *Rack1* cKO Mice

**FIGURE 2:**
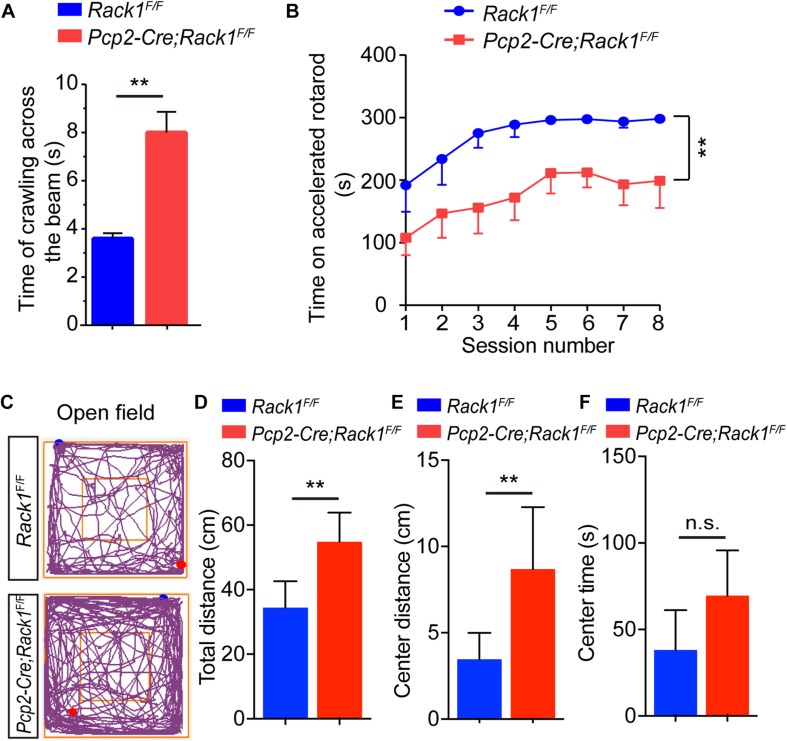
*Pcp2-Cre*;*Rack1*^*F/F*^ mutant mice show impaired motor coordination and hyperactivity. **(A)** Balanced beam experiment shows impaired motor balance in *Pcp2-Cre*;*Rack1*^*F/F*^ mutant mice. Mean ± SEM, ^∗∗^*p* < 0.0001, *n* = 10. **(B)** Time spent on the accelerating rotarod for *Rack1*^*F/F*^ control and *Pcp2-Cre*;*Rack1*^*F/F*^ mutant mice. Mean ± SEM, ^∗∗^*p* < 0.0001, *n* = 11. **(C)** Traces of locomotor activity in *Rack1*^*F/F*^ control and *Pcp2-Cre*;*Rack1*^*F/F*^ mutant mouse in an open field test (OFT). **(D)** Averaged group data of total distance traveled in control and mutant mice in the OFT. Mean ± SEM, ^∗∗^*p* = 0.0062, *n* = 8. **(E)** Averaged group data of center distance traveled in control and mutant mice in the OFT. Mean ± SEM, ^∗∗^*p* = 0.0096, *n* = 8. **(F)** Averaged group data of the time spent in the central zone in control and mutant mice. Mean ± SEM, ^∗∗^*p* = 0.0624, *n* = 8, n.s. = not significant.

To further evaluate the effect of Rack1 knockout in PCs on locomotion, 8-week-old *Pcp2-Cre;Rack1*^*F/F*^ and control mice were selected for balance-related behavioral testing. *Pcp2-Cre;Rack1*^*F/F*^ mice did not show obvious ataxia in standard cages. However, they performed poorly, with a remarkably longer time when walking on a narrow elevated beam (Figure 2A, 3.6 ± 0.22 s in mutant vs. 8.0 ± 0.86 s in control, *p* < 0.0001, *n* = 10), which indicated that the balance ability of mutant mice was significantly decreased. In addition, the time of *Pcp2-Cre;Rack1*^*F/F*^ mice staying on the accelerating rotarod was significantly shorter than that of the control mice (Figure 2B, 211.4 ± 20.11 s in mutant vs. 298.1 ± 1.81 s in control, *p* < 0.0001, *n* = 11), indicating the deficits in fine motor coordination skills in *Pcp2-Cre;Rack1*^*F/F*^ mutants. Moreover, *Rack1* mutant mice also showed impaired motor learning, in which mutant mice exhibited declined improvement after multiple sessions on the accelerating rotarod compared with controls (Figure 2B).

In addition to motor function, the cerebellum has been implicated in various cognitive and social behaviors. The dysfunction of PCs have been observed in autism and schizophrenia (Andreasen and Pierson, 2008; Yeganeh-Doost et al., 2011; Peter et al., 2016). Thus, a series of behavioral tests were performed to assess whether Rack1 ablation in PCs would affect different domains of mouse behavioral repertoire. In the open field tests, Rack1 mutant mice exhibited dramatic hyperactivity (Figure 2C). In a limited time (5 min), the mutant mice travel more distances compared to controls (Figure 2D, 53.6 ± 8.6 cm in mutant vs. 35.2 ± 9.6 cm in control, *p* = 0.0062, *n* = 8), especially at the center zone of the place (Figure 2E, 8.3 ± 4.2 cm in mutant vs. 3.6 ± 1.9 cm in control, ^∗∗^*p* = 0.0096, *n* = 8; Figure 2F, 65.6 ± 18.6 s in mutant vs. 45.2 ± 19.4 s in control, ^∗∗^*p* = 0.0624, *n* = 8). Together, these results indicate that *Pcp2-Cre;Rack1*^*F/F*^ mice exhibit hyperactivity and a significant deficit in motor coordination.

### Decreased PF–PC Synaptogenesis in *Rack1* cKO Mice

**FIGURE 3:**
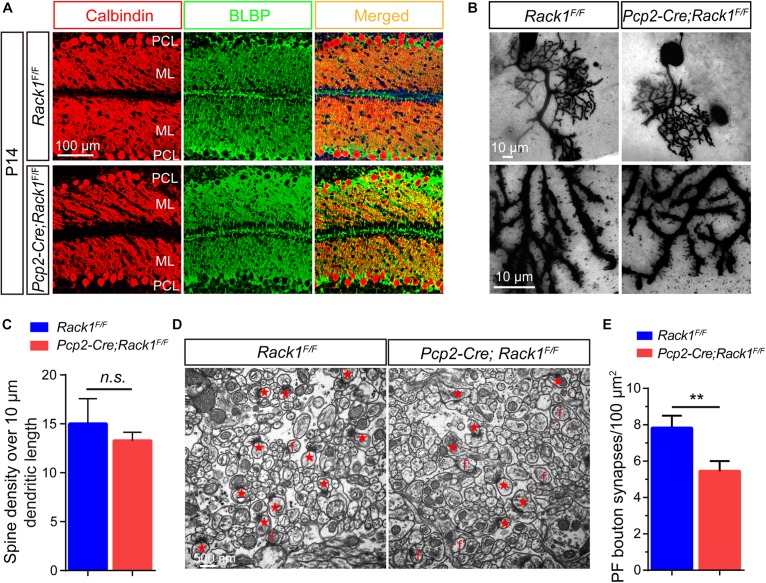
Decreased parallel fiber (PF)–Purkinje cell (PC) synaptogenesis in *Pcp2-Cre*;*Rack1*^*F/F*^ mutant mice. **(A)** Coimmunofluorescent staining of cerebellar sections with anti-Calbindin and anti-BLBP antibodies displayed the normally polarized distribution of PCs in *Pcp2-Cre*;*Rack1*^*F/F*^ mutant mice compared to control littermates. Scale bar = 100 μm. **(B)** The morphology of dendritic spines of PCs in control and mutant mice was illustrated by Golgi staining. Scale bar = 10 μm. **(C)** The statistical result shows no significant difference between *Rack1*^*F/F*^ control and *Pcp2-Cre;Rack1^*F/F*^* mutant mice in terms of the density of PC dendritic spines. Mean ± SEM, *p* = 0.1878, *n* = 5, n.s. = not significant. **(D)** Representative electron micrographs of the molecular layer within the cerebellar cortex from *Rack1*^*F/F*^ control and *Pcp2-Cre*;*Rack1*^*F/F*^ mutant mice at P21. Synapses comprising of presynaptic PF terminal boutons opposed to postsynaptic PC spines are marked with red asterisks. Free spines and mismatched synapses are indicated by f. Scale bar = 500 nm. **(E)** Quantification of the density of PF–PC synapses shows the reduction in *Pcp2-Cre*;*Rack1*^*F/F*^ mutant mice compared to that of the control *Rack1*^*F/F*^ littermates. Mean ± SEM, ^∗∗^*p* < 0.001.

Moreover, immunofluorescence staining was employed to identify PCs and Bergmann glial cells using Calbindin and BLBP antibodies, respectively, to analyze the fine lamination and morphological differences in mutant cerebellum. As was shown in Figure 3A, in the mutant cerebellum, the Calbindin^+^ PCs and BLBP^+^ Bergmann glial cells were both well and neatly organized, with a single layer of polarization distributed in the PC layer, suggesting the normal cerebellar cortex stratification in Rack1 mutant mice. Nevertheless, due to the excessive number of Calbindin^+^ PCs in the brain slices, it was almost impossible to accurately count the number of dendritic branches and dendritic spines of PCs. Therefore, the Golgi staining method was adopted to sparsely illustrate the morphology of PCs. In general, it showed that there is no obvious distinction of dendritic branches and spine density in the PCs between control and Rack1 mutant mice (Figure 3B). Statistical results also confirmed that dendritic spine density was comparable to that of control littermates (Figure 3C, 13.3 ± 0.39/10 μm in mutant vs. 15.0 ± 1.15/10 μm in control, *p* = 0.1878, *n* = 5). Thus, specific deletion of Rack1 in PCs does not impair its morphogenesis and cytoarchitecture.

Owing to the fact that PC dendrites could convert excitatory PF input from granule cells into signals, they play an important role in synaptic plasticity and motor learning (Rowan et al., 2018). Next, we asked whether the impaired motor coordination in Rack1 mutant mice was caused by the synaptogenesis deficiency or synaptic dysfunction. Therefore, we assessed the effect of Rack1 ablation in PCs on the synapse formation between PCs dendrites and PFs from granule cells by electronic microscope analysis. As shown in Figure 3D, the black high-density postsynaptic materials marked by the red asterisks was the excitatory synapse formed by PCs and PFs. Quantitative analysis shows that the density of presynaptic PF boutons in Rack1 mutant mice was substantially lower than that of control (Figure 3E, 5.4 ± 0.56/μm^2^ in mutant, *n* = 17, vs. 7.8 ± 0.69/μm^2^ in control, *n* = 22, *p* < 0.001). We also found there were more free or mismatched spines in Rack1 mutant cerebellum, suggesting the defective synaptogenesis in mutant mice. Together, these ultrastructural results suggest that Rack1 is able to promote synaptogenesis between PC dendrites and PFs in the cerebellar cortex.

### Decreased Synaptic Transmission and Impaired LTD in *Rack1* cKO Mice

**FIGURE 4:**
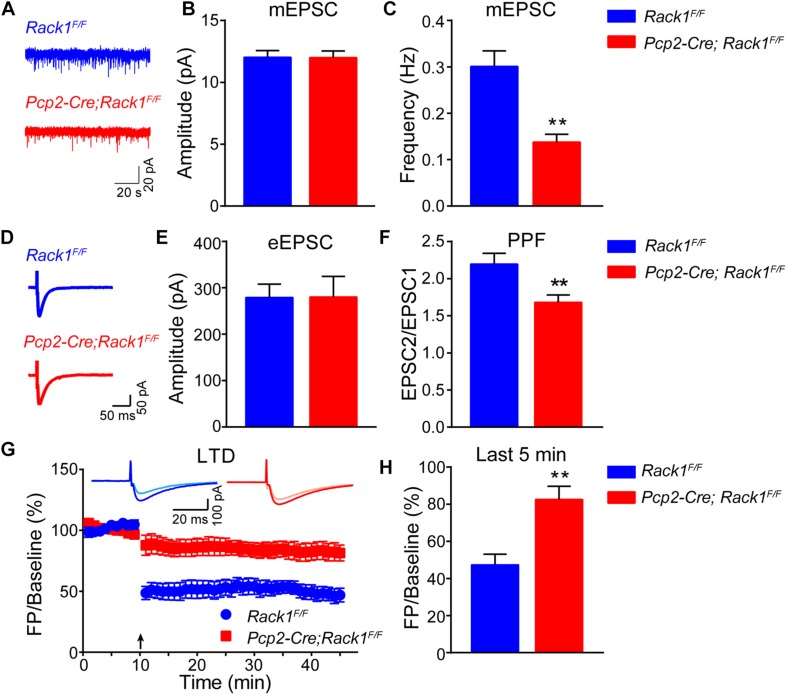
Altered synaptic transmission and long-term depression (LTD) in *Pcp2-Cre*;*Rack1*^*F/F*^ mice. **(A)** Representative traces of miniature excitatory postsynaptic currents (mEPSCs) recorded in the presence of 1 μM tetrodotoxin and 0.1 mM picrotoxin (PTX). **(B)** Summary graphs of the amplitudes of mEPSCs (*Rack1*^*F/F*^: *n* = 20 cells/4 mice; *Pcp2-Cre*;*Rack1*^*F/F*^: *n* = 17/4 mice). **(C)** Summary graphs of the frequency of mEPSCs (*Rack1*^*F/F*^: *n* = 20 cells/4 mice; *Pcp2-Cre*;*Rack1*^*F/F*^: *n* = 17/4 mice). ^∗∗^*p* = 0.0078, *n* = 12. **(D)** Representative traces of action potential-evoked EPSCs recorded in 0.1 mM picrotoxin. **(E)** The graphs of the amplitudes of action potential-evoked EPSCs (*Rack1*^*F/F*^: *n* = 14/4 mice; *Pcp2-Cre;Rack1^*F/F*^*: *n* = 10/3 mice). **(F)** The graphs of paired-pulse facilitation (*Rack1*^*F/F*^: *n* = 12/4 mice; *Pcp2-Cre*;*Rack1*^*F/F*^: *n* = 12/3 mice). ^∗∗^*p* = 0.0002. **(G)** Parallel fiber-LTD in *Rack1*^*F/F*^ (filled blue circles) and *Pcp2-Cre*;*Rack1*^*F/F*^ (filled red squares) mice. Each data point represents the average of four consecutive responses evoked at every 15 s. Representative traces before (light) and after (dark) LTD induction are shown. **(H)** Summary graphs of average amplitude of parallel fiber-LTD of the last 5 min (*Rack1*^*F/F*^: *n* = 9/4 mice; *Pcp2-Cre*;*Rack1*^*F/F*^: *n* = 8/4 mice). ^∗∗^*p* = 0.0015.

The requirement for the Rack1 in synaptogenesis in the cerebellar cortex led to the postulation that synaptic transmission at PF–PC synapses might be affected in Rack1 mutants. Our electrophysiological analysis in acute cerebellar slices revealed that the amplitude of evoked EPSC at PC–PF synapses was normal between *Pcp2-Cre;Rack1*^*F/F*^ and *Rack1*^*F/F*^ mice (Figures 4A,B, 280.2 ± 44.45 pA, *n* = 10 in mutant vs. 279.2 ± 28.63 pA, *n* = 14 in control, *p* = 0.9855). However, the ratio of paired-pulse facilitation measured at an interval of 80 ms was reduced nearly 20% (Figures 4C,D, 1.7 ± 0.10 in mutant vs. 2.2 ± 0.14, *p* = 0.0078, *n* = 12), suggesting that the presynaptic glutamate release was impaired. Moreover, there was no difference in the amplitude of the presynaptic volley in mice between *Pcp2-Cre;Rack1*^*F/F*^ mutant and *Rack1*^*F/F*^ control mice, illustrating that the impairment of evoked EPSC in *Rack1* mutant mice is not due to the difference in axon excitability (data not shown).

Then, we asked whether the neurotransmission defects in *Rack1* mutant mice might be due to the secondary effect of the changed synapse numbers. As expected, the frequency of mEPSCs in PCs was reduced in acute cerebellar slices from *Pcp2-Cre;Rack1*^*F/F*^ mice compared to *Rack1*^*F/F*^ mice (Figure 4F, 0.14 ± 0.02 pA, *n* = 17 in mutant vs. 0.30 ± 0.03 pA, *n* = 20 in control, *p* = 0.0002), consistent with the previous conclusion that synapse number is reduced in *Rack1* mutants. The amplitude of mEPSCs was hardly reduced in *Rack1* knockout mice (Figure 4E, 11.99 ± 0.54 pA, *n* = 17 in mutant vs. 12.02 ± 0.55 pA, *n* = 20 in control, *p* = 0.9724), suggesting that the reactivity of the postsynaptic membrane was normal. Collectively, our electrophysiological and electronic microscope results suggest that the deficiency of synaptic transmission between PF-PCs in *Rack1* mutants might be due to the impaired synaptogenesis.

Long-term depression has been proposed as a potential factor contributing to motor learning in the cerebellar cortex (Kakegawa et al., 2018; Zamora Chimal and De Schutter, 2018). Given that *Pcp2-Cre;Rack1^*F/F*^* mutant mice showed impaired motor coordination and hyperactive locomotion, we next examined the LTD at PF–PC synapses with voltage-clamp mode both in control and *Rack1* mutants (Figure 4G). Our results showed that PCs in *Rack1*^*F/F*^ mice performed robust PF-LTD induction (Figure 4H, last 5 min, 47.4 ± 5.73% of baseline; *n* = 9) in response to repetitive PF stimulation. However, the induction of LTD in PCs was significantly impaired in *Pcp2-Cre;Rack1^*F/F*^* mutant cerebellum (Figure 4H, last 5 min, 82.6 ± 7.14% of baseline; *n* = 8, *p* = 0.0015).

### The Expression of Major Postsynaptic Components Were Not Affected in *Rack1* cKO Mice

**FIGURE 5:**
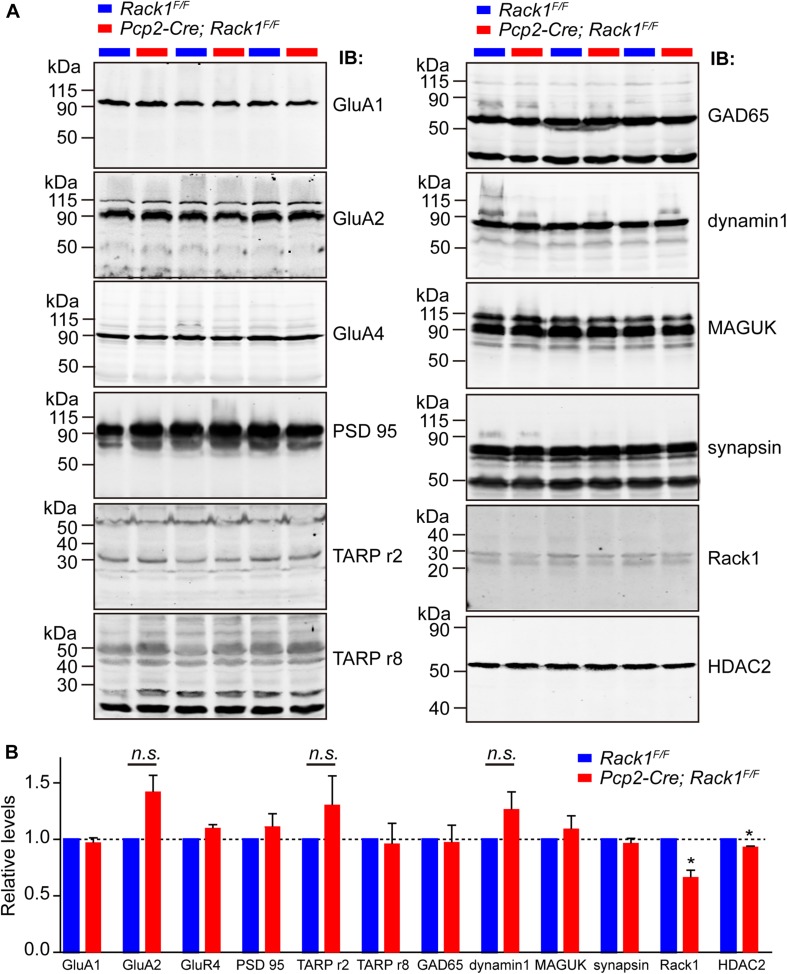
The expression of major synaptic components was not affected in *Pcp2-Cre*;*Rack1*^*F/F*^ mutant mice. **(A)** Representative Western blots examining the expression of major postsynaptic components in *Rack1*^*F/F*^ control and *Pcp2-Cre*;*Rack1*^*F/F*^ mutant cerebellum at postnatal day 21 (P21). **(B)** Quantitative analysis indicates normal levels of postsynaptic components but reduced level of HDAC2 expression in *Pcp2-Cre*;*Rack1*^*F/F*^ mutants, ^∗^*p* = 0.0092, *n* = 3. Reduced expression of Rack1 was confirmed in *Pcp2-Cre*;*Rack1*^*F/F*^ mutant compared to control littermates. Mean ± SEM, ^∗^*p* = 0.0056, *n* = 3. The expression of GluA2, TARPr2, and dynamin 1 in mutant mice is not significant compared to control littermates, *p* = 0.0962, 0.1346, and 0.1125, respectively.

Motor PF-LTD deficits may result from altered ion channel or glutamatergic-transmission-associated protein. Thus, the expression of ionotropic glutamate receptors were examined. We found that the level of AMPA-associated proteins such as GluA1/2/4 and transmembrane AMPA-receptor-regulated protein γ2/8 were not altered significantly in relation to those of PSD95 and GAD65 (Figures 5A,B, *p* > 0.05). Glutamatergic-transmission-associated protein such as membrane-associated guanylate kinase, Dynamin1, and Synapsin were also indistinguishable between *Rack1*^*F/F*^ control and *Pcp2-Cre;Rack1^*F/F*^* mutant cerebellum (Figures 5A,B, *p* > 0.05), suggesting that deletion of Rack1 in PCs has no significant effects on the expression of synaptic proteins.

Our previous work has shown that Rack1 promotes the development of granule cells via regulating the stability of HDAC1/2 in GCPs (Yang et al., 2019). Here, we also found that the expression of HDAC1/2 proteins were significantly decreased in *Pcp2-Cre*;*Rack1*^*F/F*^ mutant cerebellum compared to *Rack1*^*F/F*^ control (Figures 5A,B, 93.5 ± 1.37% in mutant vs. 100.0 ± 1.58% in control, *n* = 3, *p* = 0.0092). Interestingly, previous work has shown that HDAC1/2 together with Chd4, RbAp48, Mbd3, and Mta1/2 constitute the remodeling of nucleosome and deacetylation complex, which programs the differentiation of presynaptic sites and triggers synaptic connectivity in the PF–PCs (Yamada et al., 2014). Thus, the decreased expression of HDAC1/2 in PCs might be responsible for the malformation of PF–PCs synaptogenesis.

## Discussion

Germline deletion of Rack1 in *Drosophila* or mice both causes their death at embryonic stage (Kadrmas et al., 2007; Volta et al., 2013). Our previous work shows that deletion of Rack1 in neuronal precursor cells does not cause embryonic lethality but severe neural developmental deficiency, indicating that the early death in Rack1 null mutants is not due to brain abnormalities (Yang et al., 2019). We have shown that ablation of Rack1 in either neural stem cells or GCPs disrupts cerebellar morphogenesis (Yang et al., 2019). Here, we show that specific deletion of Rack1 in PCs in mice causes delayed formation of specific vermis lobule as well as defective motor coordination and motor learning. Given that PC-derived Shh plays a critical role in promoting the proliferation of GCPs by disinhibition of its coreceptor smoothened and activation of transcriptional factors Gli1/2 (Lee et al., 2010; Shi et al., 2014). We assume that ablation of Rack1 in PCs might caused the decreased secretion of Shh, which might delay the development of cerebellar foliation in *Pcp2-Cre;Rack1^*F/F*^* mutants. Another interesting phenotype of mutant mice was hyperactivity in the open field test. The PCs are considered the principal neurons in the cerebellar cortex which provide their sole outputs by projecting to the deep cerebellar nuclei and the vestibular nuclei (Sillitoe and Joyner, 2007). This circuit is critical in controlling the ongoing execution as well as coordinating the planning of limb movement. Previously, Waite et al. have demonstrated that selectively ablation of PCs in rats resulted in hyperactivity in open field (Waite et al., 1999). Thus, it is possible that the inhibitory output of the cerebellar cortex was reduced in *Pcp2-Cre;Rack1^*F/F*^* mutants, leading to an elevated locomotive activity.

The cerebellum is important for movement control, which is tightly regulated by precise cerebellar circuits (Fetz, 1993; Voogd and Glickstein, 1998). PCs integrate signals from two major excitatory inputs including the climbing fibers and PFs that convey signals of the inferior olive or the mossy fiber relay system, respectively (Sillitoe and Joyner, 2007). Our finding that specific ablation of Rack1 in PCs caused significantly impaired PF–PC synaptogenesis and LTD induction suggest that Rack1 is essential for the development and function of PF–PC synapses. Thus, these findings provide another interesting aspect of Rack1 function in the cerebellar circuits. Owing to the fact that PF–PC circuits play important roles in movement control, motor learning, and non-motor functions such as language, social interaction, and expectation of reward (Strick et al., 2009; Wagner et al., 2017), our studies provide a novel evidence how dysfunction of Rack1 in PCs may cause the movement and motor coordination disorders in mutant mice.

Interestingly, Rack1 has been shown to inhibit the NMDAR-mediated activity by preventing the phosphorylation of NR2B mediated by the tyrosine kinase Fyn (Yaka et al., 2002). Another interesting result of our work is that the reduction in the frequency but not the amplitude of the mEPSCs was identified in *Pcp2-Cre;Rack1^*F*/*F*^* mutant mice. Although we did not find significant changes of synaptic protein expression in the *Rack1* mutant mice, the finding that the PC-specific deletion of Rack1 selectively affected excitatory presynaptic transmission suggests an important role of Rack1 in synaptic homeostasis. Moreover, neuroadaptation is thought as one of the mechanisms that contributes to the synaptic changes in response to sustained morphine exposure (De Vries and Shippenberg, 2002), and Rack1 has been exactly implicated in this process by activation of extracellular signal-regulated kinase–cAMP response element binding signaling in hippocampus (Liu et al., 2016). However, whether this pathway is involved in Rack1-mediated PF-PC synaptic transmission is still elusive.

The climbing fiber innervation of PCs has been shown to be important for normal motor behavior in mice, and PF-mediated mGluR1 and PKCγ activation is essential for LTD and late-phase climbing fiber elimination (Kano et al., 1995, 1997; Ichise et al., 2000). The bidirectional plasticity of the PF–PC synapses has been nicely demonstrated, which shows that climbing fiber activity can reverse the PF–PC synapse long-term potentiation (LTP) into LTD (Coesmans et al., 2004). Moreover, the presynaptic LTP and LTD of PF–PC synaptic plasticity also have been elegantly demonstrated *in vitro* and *in vivo* in mice (Qiu and Knopfel, 2009; Chu et al., 2014; Wang et al., 2014; Bing et al., 2015). PKCα and CaMKII activation has been shown to be essential for PF–PC synapse LTD (Leitges et al., 2004; Hansel et al., 2006; van Woerden et al., 2009). However, how CaMKII and PKCs regulate the LTD induction is still poorly understood.

Notably, as a scaffolding protein, Rack1 serves as an intracellular receptor for activated PKC, which is responsible for subcellular localization of PKC (Ron et al., 1994). PKC has been shown to be important for AMPA receptor internalization by phosphorylation of GluR2 subunit (Chung et al., 2003). PKCα also regulates the AMPA receptor clustering by phosphorylation of GluR2 at Ser880 and thereby reducing their binding to glutamate receptor interacting proteins (Matsuda et al., 1999). Although in our study, we do not find the expression changes of GluR2 in *Pcp2-Cre;Rack1^*F/F*^* mutant mice, we cannot rule out the possibility that the phosphorylation of GluR2 is altered in the absence of Rack1 in PCs. Therefore, the impaired motor coordination and PF–PC synaptic transmission in PC-specific Rack1 knockout mice is presumably mediated by the deficiency in Rack1-dependent synaptic plasticity. In addition, LTP but not LTD at PF–PC is also known to be related to cerebellar motor learning (Schonewille et al., 2011; Gao et al., 2012). Previous study shows that PF–PC synaptic plasticity is regulated by a kinase/phosphatase switch. Protein phosphatase 1 (PP1), PP2A, and PP2B (calcineurin) play important roles in PF–PC LTP (Belmeguenai and Hansel, 2005; Schonewille et al., 2010). Additional studies will be necessary to fully elucidate the role of Rack1 in PF–PC LTP/LTD, at least in part because of its influence on the presynaptic function of PF–PCs and its relative roles in the regulation of NMDAR in PCs.

It should be noted that the synaptic plasticity in the cerebellum is not only observed for the excitatory PF–PC synapses but also for inhibitory synapses formed by basket and stellate interneurons with PCs in the molecular layer (Sillitoe and Joyner, 2007). In this study, we mainly focus on the impact of Rack1 on the PF–PCs synaptogenesis and synaptic transmission. Given that GABAergic synapses of PCs is also important for cerebellar motor learning (Tanaka et al., 2013; Hirano and Kawaguchi, 2014), it would be of interest to investigate whether Rack1 is also involved in the regulation of GABA receptor activity in PCs in the near future.

## Data Availability Statement

The raw data supporting the conclusions of this manuscript will be made available by the authors, without undue reservation, to any qualified researcher.

## Ethics Statement

The animal study was reviewed and approved by the Institutional Animal Care and Use Committee of Beijing Institute of Basic Medical Sciences.

## Author Contributions

HW designed the research and wrote the manuscript. HY, CY, QZ, MW, YL, JC, FL, and YW performed the research. JZ and CZ contributed the reagents, mice, and analytic tools. CZ and HW analyzed data.

## Conflict of Interest

The authors declare that the research was conducted in the absence of any commercial or financial relationships that could be construed as a potential conflict of interest.
